# Supplementary material for: Determination of Five Sit-to-Stand Test Performance at Discharge of Stroke Patients
Source: Diagnostics (Basel). 2024 Feb 29;14(5):521. doi: 10.3390/diagnostics14050521 (PMC10930600; doi:10.3390/diagnostics14050521)
Supplement: Supplementary file 1 [file diagnostics-14-00521-s001.zip › diagnostics-2858610-supplementary.pdf]

Table S1: Linear regression covariates for the 5-STS test at discharge.

Variables	Univariate Models	
	**B (95%CI)**	**P-value**
Demographics		
Age, y	0.102(0.01-0.18)	0.019
Male	-0.849(-3.17-1.47)	0.468
Stroke characteristics		
Side affected (right)	0.622(-1.67-2.92)	0.589
Type of stroke (ischemic)	1.10(0.32-3.80)	0.871
Time from stroke to admission PT, days	0.068(0.03-0.10)	<0.001

5-STS indicates five-repetition sit-to-stand; PT, physical therapy, CI, confident interval.

Table S2. Lineal regression covariates with changes in the 5-STS test at discharge.

Variables	Univariate Models with T0 measure		Univariate Models with T1 measure	
	**B (95%CI)**	**P-value**	**B (95%CI)**	**P-value**
Demographics				
Age, y	0.058(-0.04-0.15)	0.253	0.016(-0.03-0.06)	0.540
Male	0.852(-1.82-3.52)	0.525	0.709(-0.64-2.05)	0.297
Stroke characteristics				
Side affected (right)	-0.469(-3.18-2.16)	0.723	-0.122(-1.46-1.22)	0.857
Type of stroke (ischemic)	-1.67(-4.68-1.34)	0.271	-0.127(-1.67-1.42)	0.870
Time from stroke to admission PT, days	-0.006(-0.05-0.03)	0.801	-0.007(-0.03-0.01)	0.504

5-STS indicates five-repetition sit-to-stand; PT, physical therapy, CI, confident interval.
